# Optimising Exome Prenatal Sequencing Services (EXPRESS): a study protocol to evaluate rapid prenatal exome sequencing in the NHS Genomic Medicine Service

**DOI:** 10.3310/nihropenres.13247.1

**Published:** 2022-02-03

**Authors:** Melissa Hill, Sian Ellard, Jane Fisher, Naomi Fulop, Marian Knight, Mark Kroese, Jean Ledger, Kerry Leeson-Beevers, Alec McEwan, Dominic McMullan, Rhiannon Mellis, Stephen Morris, Michael Parker, Dagmar Tapon, Emma Baple, Laura Blackburn, Asya Choudry, Caroline Lafarge, Hannah McInnes-Dean, Michelle Peter, Rema Ramakrishnan, Lauren Roberts, Beverly Searle, Sarah L. Wynn, Wing Han Wu, Lyn S. Chitty

**Affiliations:** 1 NHS North Thames Genomic Laboratory Hub, Great Ormond Street Hospital for Children, London, UK; 2 Genetics and Genomic Medicine, UCL Great Ormond Street Institute of Child Health, London, UK; 3 Exeter Genomics Laboratory, Royal Devon and Exeter NHS Foundation Trust, Exeter, UK; 4 Institute of Biomedical and Clinical Science, College of Medicine and Health, University of Exeter, Exeter, UK; 5 Antenatal Results and Choices, London, UK; 6 Department of Applied Health Research, University College London, London, UK; 7 National Perinatal Epidemiology Unit, University of Oxford, Oxford, UK; 8 PHG Foundation, University of Cambridge, Cambridge, UK; 9 Alström Syndrome UK, Torquay, UK; 10 Department of Obstetrics and Gynaecology,, Nottingham University Hospitals NHS Trust, Nottingham, UK; 11 West Midlands Regional Genetics Service, Birmingham Women's and Children's NHS Foundation Trust, Birmingham, UK; 12 Department of Public Health and Primary Care, University of Cambridge, Cambridge, UK; 13 The Ethox Centre, Nuffield Department of Population Health and Wellcome Centre for Ethics and Humanities, University of Oxford, Oxford, UK; 14 Centre for Fetal Care, Queen Charlotte's and Chelsea Hospital, Imperial College Healthcare NHS Trust, London, UK; 15 Peninsula Clinical Genetics Service, Royal Devon and Exeter NHS Foundation Trust, Exeter, UK; 16 Manchester University NHS Foundation Trust, Manchester, UK; 17 School of Human and Social Sciences, University of West London, London, UK; 18 Genetic Alliance UK, London, UK; 19 Unique - Rare Chromosome Disorder Support Group, Oxted, UK

**Keywords:** prenatal exome sequencing, genomic medicine service, ethics, counselling, study protocol, mixed methods

## Abstract

**Background:**

Prenatal exome sequencing (ES) for the diagnosis of fetal anomalies has been implemented nationally in England through the NHS Genomic Medicine Service that is based around seven regional Genomic Laboratory Hubs (GLHs). Prenatal ES has the potential to significantly improve NHS prenatal diagnostic services by increasing genetic diagnoses and informing prenatal decision-making. Prenatal ES has not previously been offered routinely in a national healthcare system and there are gaps in knowledge and guidance.

**Methods:**

We are conducting a mixed-methods evaluation of the NHS prenatal ES service. Study design draws on a framework developed in previous studies of major system innovation and Normalisation Process Theory. There are five interrelated workstreams. Workstream-1 will use interviews and surveys with professionals, non-participant observations and documentary analysis to produce in-depth case studies at all GLHs. Data collection at multiple time points will track changes over time. In Workstream-2 qualitative interviews with parents offered prenatal ES or with previous experience of fetal anomalies will explore experiences and establish information and support needs. Workstream-3 will analyse data from all prenatal ES tests for nine-months to establish service outcomes (e.g. diagnostic yield, referral rates, referral sources). Comparisons between GLHs will identify factors (individual or service-related) associated with any variation in outcomes. Workstream-4 will identify and analyse practical ethical problems. Requirements for an effective ethics framework for an optimal and equitable service will be determined. Workstream-5 will assess costs and cost-effectiveness of prenatal ES versus standard tests and evaluate costs of implementing an optimal prenatal ES care pathway. Integration of findings will determine key features of an optimal care pathway from a service delivery, parent and professional perspective.

**Discussion:**

The proposed formative and summative evaluation will inform the evolving prenatal ES service to ensure equity of access, high standards of care and benefits for parents across England.

## Plain English summary

Background: Prenatal exome sequencing is a new test that is offered through the NHS Genomic Medicine Service. Prenatal exome sequencing is offered to pregnant women when ultrasound scans suggest that their baby may have a genetic condition that cannot be diagnosed using standard tests. If a genetic condition is diagnosed this can give parents important information about the outlook for their baby. It can also help with their decisions about whether to continue or end the pregnancy, pregnancy management, post-birth care and future pregnancies.

Study methods: The aim of this study is to evaluate the prenatal exome sequencing service.

To do this we will;

1. Study how prenatal exome sequencing is delivered across England using surveys and interviews with professionals.
2. Interview parents to ask what they think of prenatal exome sequencing and how support and information could be improved
3. Look at how many parents have prenatal exome sequencing and the test results. We will look carefully at who has access to the test and whether any particular groups are less likely to be offered testing.
4. Conduct workshops with health professionals and parents to identify any practical or ethical problems that arise when prenatal exome sequencing is offered.
5. Look at the cost of prenatal exome sequencing and compare it to the cost of other tests that are offered to diagnose genetic conditions in pregnancy.
6. Gather our findings together to make recommendations for best practice.

Patient and Public Involvement: A patient and public Involvement, engagement and participation (PPIEP) advisory group will work closely with the research team to design the study and develop study materials. They will also help us understand our findings to make sure the information and recommendations that come out of our research will be helpful to parents and the NHS.

## Introduction

Fetal anomalies occur in approximately 2–5% of pregnancies and cause around 20% of perinatal deaths ^1, 2^. When fetal structural anomalies are detected by ultrasound, routine prenatal testing options can include karyotyping, chromosomal microarray or gene-specific panels, which will diagnose around 40% of cases. Prenatal exome sequencing (ES), which can interrogate multiple genes at high resolution in a single test, has been shown to improve diagnostic yields by 8–10% in unselected pregnancies where there is a structural abnormality and normal karyotype and chromosomal microarray ^3, 4^. Factors such as the rigour of eligibility criteria, testing platforms, trio (parents and fetus) versus singleton (fetus only) sequencing and, in particular, whether there has been selection following genetic review all impact on diagnostic yield ^5^. A growing number of studies have demonstrated the clinical utility of prenatal ES ^6– 8^ and recent guidelines from professional bodies have considered the evidence for the use of this test ^9– 11^. Accurate genetic diagnosis allows tailored parental counselling about prognosis; informs decision-making about pregnancy management; and aids planning for delivery and perinatal management. It also circumvents the pre- and postnatal ‘diagnostic odyssey’ and allows accurate counselling about recurrence risk for future pregnancies. 

The NHS in England is the first national healthcare system to systematically embed genome and exome sequencing in routine clinical care. To do this, genetic services across England have been reconfigured to establish a national NHS Genomic Medicine Service (GMS) which consolidates all genomic testing into a unified service that is delivered through seven regional NHS Genomic Laboratory Hubs (GLHs) and NHS Genomic Medicine Service Alliances (GMSAs) with a National Genomic Test Directory which dictates which genomic tests are available through this service ^12^. The NHS GMS aims to deliver high throughput and high-quality genomic testing with equity of access for patients across the NHS ^13^. Prenatal ES was implemented nationally in the NHS GMS in October 2020 and is offered to parents across England when anomalies identified on fetal ultrasound are considered likely to have a genetic aetiology, as determined by a multidisciplinary team that includes a clinical geneticist. Prenatal ES is listed as R21 in the National Genomic Test Directory ^12^. 

Professional bodies have highlighted the many practical considerations to implementing a service that delivers prenatal ES ^9– 11^. As prenatal ES is being implemented nationally in England, there is the potential for wide variation in referrals, uptake and diagnostic rates. Research studies considering parent or professional views on prenatal ES largely support offering prenatal sequencing but raise concerns over the potential for increased parental anxiety, informed consent, management of parent expectations, cost, which results to report and when to reinterpret results ^14– 19^. The need for health professional education and new approaches to genetic counselling that support informed choice during a distressing and time-pressured period have also been highlighted ^15, 18^. Another key challenge will be counselling parents around the range of findings and possible uncertainties ^20^. As a result, it is crucial that the prenatal ES service is evaluated and guidelines developed to support high quality care for parents and facilitate delivery of an equitable and efficient national service.

Here we provide an outline of the optimising EXome PREnatal Sequencing Services (EXPRESS) study; a three-year prospective evaluation of prenatal ES in the NHS GMS. The EXPRESS study will analyse the national implementation of prenatal ES in order to determine an optimal care pathway that maximises benefits for parents while optimising use of NHS resources. This research will capture the perceptions of parents and professionals, identify ethical and practical issues and highlight any unintended consequences of the new care pathways. As our research will commence in the first year of the prenatal ES service, we have proposed a formative evaluation that will deliver lessons for the developing service within the timeframe of the study.

## Protocol

### Study design

**Figure 1.**
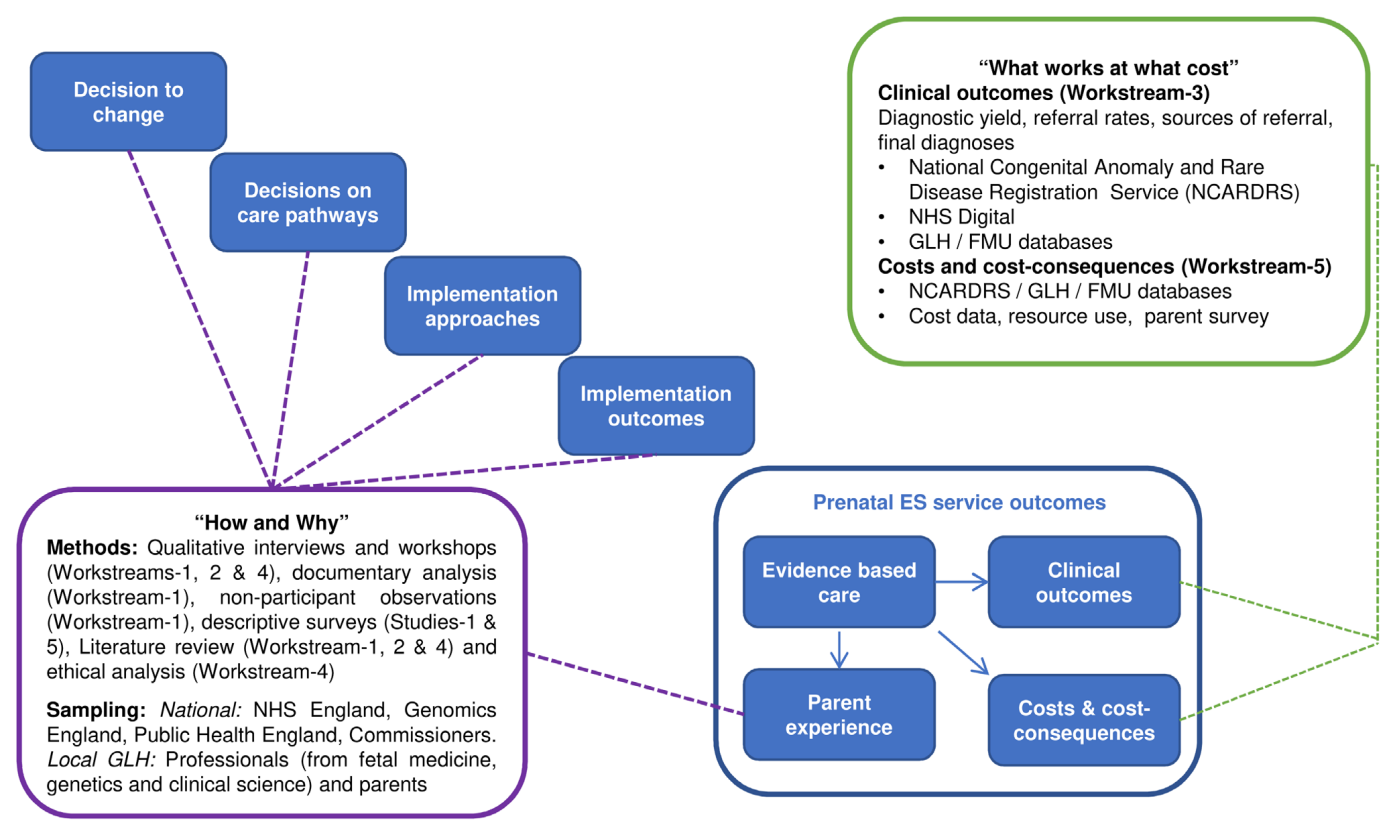
Conceptual framework underpinning our evaluation of the prenatal ES service. Adapted from Fulop *et al.* ^21, 22^.

EXPRESS is a multi-site, mixed-methods study that will evaluate how prenatal ES is offered in the NHS GMS. We will combine qualitative analyses of the service, stakeholder perspectives and ethical considerations with quantitative analyses of clinical outcomes and cost effectiveness. The research design draws on a framework developed in previous studies of major system innovation, which highlighted the key processes: the decision to change, developing and agreeing new service models, how changes are implemented, and implementation outcomes ^21, 22^. Our evaluation of the outcomes of the prenatal ES service (what works and at what cost) will be grounded in an understanding of the planning and implementation of the service (how and why) ( Figure 1). In addition, as this is the first time prenatal ES will be implemented systematically across a national healthcare setting – and on such a large scale – we will draw on Normalisation Process Theory ^23, 24^, which emphasises agency, cooperation and coordination in a social system as key elements in the successful embedding of a complex intervention.

### Study oversight

A Steering Committee with academic, professional and patient and public involvement, engagement and participation (PPIEP) members and a PPIEP Advisory Group will oversee the evaluation, providing guidance and feedback through regular interactions with the research team throughout the study.

### Critical distance

Our research team includes several clinicians and laboratory scientists with a professional role in the NHS GMS, whose expertise will be crucial throughout the study. NJF and SM are independent of the NHS GMS and have extensive experience in the evaluation and appraisal of healthcare services and they will be responsible for ensuring that a “critical distance” is maintained throughout our evaluation. 

### Patient and public involvement, engagement and participation

We are embedding PPIEP in all aspects of our study. Patient advocates are co-applicants on the grant and a PPIEP Advisory Group has been formed that includes representatives of rare condition charities and members who can advise on including the views of ethnic minority groups. The PPIEP Advisory Group are inputting into the design of the study and the development of study materials for parents. They have reviewed and revised parent-facing documents such as participant information sheets and topic guides and advised on plans for the recruitment of parents for qualitative interviews. Research findings will be shared with the PPIEP Advisory Group throughout the study and they will support the development of recommendations and information resources that will be helpful to parents, families and the NHS. Another key element of our PPIEP strategy is to have a qualitative researcher embedded within the parent support group Antenatal Results and Choices (ARC) who will have a broad appreciation of the information and support needs of parents who have experienced anomalies in pregnancy.

### Study aims and objectives

The aim of EXPRESS is to provide a formative and summative mixed-methods evaluation of the new prenatal ES service, to ensure national delivery of an equitable, acceptable, ethical, robust and cost-effective care pathway that improves the quality of care for parents undergoing prenatal diagnosis in fetuses with anomalies likely to have a genetic aetiology.

Specific objectives:

A. Determine the clinical care pathways for prenatal ES in each of the seven GLHs.
B. Establish whether prenatal ES is understandable and acceptable to key stakeholders.
C. Identify the education and information needs of parents and health professionals, and how they are best addressed.
D. Establish the outcomes (diagnostic yield, referral rates, sources of referral, final diagnoses) of the prenatal ES programme, compare these between regions, and identify any factors (individual or service-related) associated with variation in outcomes.
E. Identify any new ethical issues arising from offering the prenatal ES programme in the NHS and explore how health professionals can best be supported in addressing them.
F. Formally evaluate the cost and cost-effectiveness of implementing the optimal prenatal ES pathway.
G. Determine the key features that constitute the optimal prenatal ES pathway from a service delivery, patient and professional perspective.

### Study setting

This is a nationwide study that will look at provision of prenatal ES across England through the NHS GMS. Prenatal ES is being performed through two of the seven GLHs (NHS North Thames GLH and NHS Central and South GLH). Parents will be referred through fetal medicine units (FMUs) by clinical geneticists from all GLHs/GMSAs. As such, the setting for our research will be all seven of the GLHs and their linked clinical genetic services and FMUs. The seven GLHs are; NHS Central and South GLH, NHS East GLH, NHS North West GLH, NHS North Thames GLH, NHS South East GLH, NHS South West GLH and NHS North East and Yorkshire GLH.

### Workstream overview

Our mixed-methods evaluation of the new prenatal ES service comprises five interrelated workstreams.

***Workstream-1: Defining clinical care pathways***

**Phase 1: Understand the goals and challenges for the current service**

In the first 6 months of EXPRESS we will use three approaches to gain an understanding of the anticipated goals and early challenges for the prenatal ES service.

1) To identify key challenges for service delivery we will conduct a mixed-methods systematic literature review on the use of prenatal ES in both research and clinical settings worldwide. The review will be conducted according to PRISMA guidelines ^25^.
2) To explore the drivers of implementation and examine the overarching ambitions and potential challenges for the service we will conduct 8–10 interviews at a national level with key staff involved in establishing the prenatal ES service. We will also undertake a documentary analysis and collect any available business case and policy documents relating to the implementation of prenatal ES.
3) To gather the views of professionals involved in delivering the prenatal ES service across England, we will conduct qualitative interviews with 2–3 professionals from each GLH. Invitations will be sent to professionals from a range of backgrounds including clinical genetics, fetal medicine and clinical scientists. The interviews will explore professionals’ expectations, perceptions of current challenges for delivery, foreseen ethical problems and plans for developing the service.

**Phase 2: Establish emergent care pathways and produce an overview description of services**

In months 6–18 of the study, we will produce a taxonomy of the care pathways emerging in practice for all seven GLHs. This work will document early indications of consensus and variation in service delivery, organisation and design, and will form the foundation for understanding why the different networks vary in service provision (if they do). To do this, we will conduct a cross-sectional survey with ~100 clinical and laboratory staff across England to determine how eligibility criteria are applied, consider information available to clinicians (such as high-quality ultrasound scans for phenotyping), and explore training and education needs and overall views on prenatal ES and how it is delivered. We will also examine referral pathways and patient flow from general maternity units to FMUs to genetics services. A sub-set of survey participants from a range of backgrounds and geographies will be contacted to take part in a follow-up interview that will probe their responses to the survey in more depth. In addition, to examine how processes then change over time we will monitor service delivery through 6 monthly calls with a key contacts to ask a standardised list of questions.

**Phase 3: In-depth case studies**

We will produce an in-depth case study of prenatal ES services for each of the seven GLHs. We will refer to MRC guidance ^26^ on the conduct of process evaluations for studying the implementation of complex health interventions and Normalisation Process Theory ^23, 24^ to explain how the new prenatal ES services have developed over time, and across different contexts. As the prenatal ES service is entirely new to the NHS there is no baseline, so case studies will address how the service is being delivered against service objectives, aspirations and adaptations, and the plans identified by professionals in Phase 1 and 2. We will use a case study approach ^27– 29^ to data collection. Qualitative data will be collected from semi-structured interviews with ~35 staff from a range of backgrounds, key documents, non-participant observations of relevant team meetings in each GLH and two focus groups with health professionals.

**Recruitment of professionals**

To recruit participants to semi-structured interviews and the survey, professionals from relevant backgrounds will be identified by the research team with the help of key contacts at each GLH. We will purposively sample health professionals from a range of backgrounds including clinical geneticists, genetic counsellors, fetal medicine consultants, midwives, clinical scientists and hospital chaplains. An invitation email along with a participant information sheet describing the purpose of the study will be emailed to potential participants. For non-participant observations we will notify the attendees in advance of the meeting of our intention to observe the meeting and obtain consent at the time of the meeting.

**Data collection and analysis**

Interviews will be carried out by phone, video call or face-to-face. Interviews will be digitally recorded and professionally transcribed verbatim. All qualitative data (interviews, observations, fieldwork notes, survey responses (open-ended questions and comments) and documents) will be anonymised and then analysed using the principles of codebook thematic analysis ^30, 31^. Data analysis will combine inductive and deductive approaches ^32^ as themes will be drawn from the literature and emerge from the empirical data. Data will be coded into meaningful units of text and then grouped into broader thematic categories that will be progressively reviewed and redefined. Qualitative data will be managed using NVivo version 12 (QSR International, Pty Ltd). To ensure the validity and rigour of the analysis two experienced qualitative researchers will conduct the analysis, following recommended protocols ^33^. To strengthen the credibility of the findings and include the perspectives of parents and clinicians from a range of backgrounds, themes will be reviewed and discussed with the wider research team and the PPIEP Advisory Group. Frequencies will be used to summarise findings from the quantitative survey data.

***Workstream-2: Parental views and experiences of prenatal ES.*** Parent views and experiences of prenatal ES will be gathered through qualitative interviews with at least 35 parents offered prenatal ES (recruited through FMUs) and 20 parents who have previous experience of fetal anomalies (recruited through parent support groups). Participants will be purposefully sampled to ensure there is maximum variation in terms of clinical experiences and socio-demographic factors such as ethnicity and socio-economic background.

Using a semi-structured topic guide (developed with the feedback from the PPIEP Advisory Group), we will explore parents’ views of prenatal ES and their thoughts on the information and support needs of parents. For parents offered ES, we will also ask about their experiences of the service, including what genetic counselling they received, their decision-making, motivations for having or declining testing, and costs incurred.

**Recruitment of parents offered prenatal ES**

Invitations to parents to take part in an interview will only be given after the parents have been offered ES and have made their decision to accept or decline testing and, as such, this research will not impact on their decision-making about this test. The clinical team at FMUs that have offered prenatal ES will identify parents that accepted or declined prenatal ES. A letter explaining the interview study and the Participant Information Sheet will be sent to potential participants. The letter will include an invitation to participate in an interview and they will be asked to contact the research team via telephone or email if they are interested in participating. As this will be a stressful and emotional time for parents, the researcher conducting the interviews will be guided by the clinical team as to the best time to send the letter to the parents.

**Recruitment of parents with previous experience of fetal anomalies**

We will recruit parents with previous experience of fetal anomalies (with and without experience of prenatal ES) through registered parent support groups such as ARC. Parent support group members will be invited to participate through an advertisement on the parent group website or through social media (Facebook/Twitter). Parents will be asked to contact the research team if they are interested in participating. Parents will be sent the participant information sheet and invited to ask questions about the study and make a time for the interview

**Data collection and analysis**

Interviews will be carried out by phone, video call or face-to-face at a location convenient to the participant, such as their home or an office at the recruiting hospital. Interviews will be digitally recorded, professionally transcribed, anonymised and analysed using the principles of codebook thematic analysis ^30, 31^ as described for Workstream-1 above. Our recruitment target of approximately 50 interviews is guided by our previous research focused on new approaches to prenatal testing and should be sufficient to include parents with a range of clinical experiences and socio-demographic factors ^34, 35^.

***Workstream-3: Factors associated with variation in outcomes across the GLHs.*** In this workstream we will establish the outcomes (diagnostic yield, referral rates, sources of referral, final diagnoses) of the prenatal ES service over a nine month period. These outcomes will then be compared across regions to identify any factors (individual or service-related) associated with variation in outcomes between GLHs. At the point of being consented for prenatal ES, parents will be asked to allow their data to be used for research purposes. Data will be collected from testing GLHs and will include pregnancy-level information on socio-demographics (age, socioeconomic status (Index of Multiple Deprivation, IMD) based on women’s area of residence, ethnicity), gestation at referral for testing and results of ES. Cases will be identified from GLHs and extracted for one year. Socio-economic status (Index of Multiple Deprivation, IMD) based on women’s area of residence will be determined from postcodes obtained from GLH records.

**Data collection and analysis**

Pregnancy outcome data will be sourced from FMUs on all women referred for prenatal ES. Pregnancy outcomes will be validated though collaboration with the National Congenital Anomaly and Rare Disease Registration Service. Data will be obtained at the pregnancy level on all women giving birth in England over the same time-period from NHS Digital, and linked within NHS Digital on the basis of women’s NHS number to the FMU outcome data before analysis of an anonymised dataset. Multi-level models will then be built examining the influence on outcomes of individual and GLH level factors (based on network pathways identified in Workstream-1).

Descriptive analyses: The following information will be described for each GLH:

- Number of women giving birth in the GLH area annually (mapped on the basis of births in referring units and their associated home births).
- Characteristics of women giving birth in each GLH area: Age (mean, SD), IMD score (% in each quintile), ethnicity (grouped according to UK census classification).
- Number of women referred for prenatal ES annually.
- Characteristics of women referred for prenatal ES in each GLH area: Age (mean, SD), IMD score (% in each quintile), ethnicity (grouped according to UK census classification), source of referral, final diagnosis made, gestation at diagnosis (median, IQR) and pregnancy outcome (termination, pregnancy loss, live birth, stillbirth).

Other characteristics of each GLH will have been described as part of Workstream-1 and are likely to include categorical factors such as case selection; links between FMUs, clinical genetics and laboratories; laboratory pipelines; turn-around times; and interpretation and reporting of results.

Overall referral rates with 95% confidence intervals in each GLH will be calculated, and referral rates within population subgroups (IMD quintiles, ethnic groups) calculated to assess equity across the system and ensure the needs of ethnic minority and seldom heard populations are being appropriately considered. Factors associated with variations across GLHs in referral rates (population characteristics, GLH factors) will be examined using regression analysis. Similarly, in each GLH diagnostic yield will be calculated (proportion of women with a clear final diagnosis on the basis of prenatal ES) as well as outcomes of prenatal ES (proportion of women undergoing ES opting for termination, live birth rate, stillbirth rate and proportion of births with a confirmed anomaly) and factors associated with variation examined.

***Workstream-4: Ethical analysis.*** To inform and promote the achievement of high ethical standards in the NHS GMS, we will analyse ethical issues arising in the delivery of prenatal ES, through an ethical analysis of stakeholder workshops, the interviews with professionals (Workstream-1), interviews with parents (Workstream-2), and engagement with the PPIEP Advisory Group. Ethical issues to address are likely to include, but will not be limited to, the following:

- Enabling adequate levels of informed consent for this complex testing
- Equity of access
- Decisions about reporting results to parents in the context of increased uncertainty and complex probabilities
- Questions relating to the sharing of data: for clinical and/or research purposes
- Clarification of the nature and scope of the duties of care of health professionals and laboratory staff when offering this complex testing to pregnant women

A systematic scoping review of the relevant literature, professional guidelines and reports of advisory bodies on the prenatal uses of genomics and genetics will provide an initial mapping of the likely ethical issues and themes for further investigation. Themes will be incorporated into interviews with professionals (Workstream-1) and parents (Workstream-2). Results will be combined to inform a comprehensive analysis of core ethical concepts and considerations to aid development of a draft ethics framework, which will be revisited and revised in light of findings from other arms of the study and three-four ethics workshops. The workshops will bring together clinical and laboratory staff from across the seven GLHs and associated clinical services, the PPIEP Advisory Group and patient groups. The workshops will gather evidence about ethical problems arising in practice and explore perspectives on the nature and scope of professional responsibilities in the provision of prenatal ES. The workshops will allow us to gather a rich account of the ethical aspects of implementation in practice and identify possible solutions and/or forms of effective ethical advice. We will map key issues, explore themes in-depth and seek views on requirements for an effective ethics framework.

***Workstream-5: Health economic evaluation***

**Phase 1: Cost of prenatal ES versus standard testing**

We will undertake a detailed micro-costing exercise to evaluate the unit costs of prenatal ES and other tests at each GLH. This will provide evidence on the likely affordability of prenatal ES for use in routine care. Micro-costing is a highly detailed costing approach that identifies all the underlying resources required for an intervention/activity, such as equipment, consumables, and staff time, and then calculates costs for these resources. We will follow a previously used approach to costing genetic tests ^36^. The standard operating procedures for each test will be used to develop costing questionnaires to collect the resource use information. The questionnaires will cover each stage in the experimental protocol from sample preparation to data interpretation and reporting. Resource use information on staff time, consumables, and equipment will be derived from the questionnaires. The analysis will account for the expected cost of any errors or failures during the testing processes. For capital equipment items, the cost will be spread over the item's predicted lifetime and depreciated using equivalent annual costing. The cost of staff and consumables will be taken from market prices. The cost per test will be based on the measured annual throughput of the sequencing platforms. For standard testing we will adopt a two-stage approach. As these tests are currently established in routine care we will ascertain if each GLH has carried out their own micro-costing analysis for reimbursement purposes – in previous similar studies we have found this to be the case. If so, we will use these costs for our analysis, ensuring that the cost components included are commensurate across GLHs. If this is not the case, then we will undertake our own micro-costing exercise at each GLH where costs of standard tests are not available, utilising the same approach as described above for ES. Due to the sensitivity of these data the results for each individual GLH will remain anonymous and we will present mean and (anonymised) ranges only.

**Phase 2. Costs and consequences of the optimal prenatal ES pathway**

We will undertake cost and cost consequences analyses of the different delivery pathways at each of the seven GLHs, plus the identified optimal prenatal ES pathway. In previous research we have argued that quality-adjusted life years are not commonly used in economic evaluations of prenatal testing for fetal anomalies ^37^, and therefore we will not use them here (nor undertake a cost-utility analysis). Costs will be estimated from the perspectives of both the NHS and families, with the time horizon being the duration of pregnancy. Using an approach we have used in similar studies ^37, 38^, the analysis will proceed in the following stages:

1) We will delineate the pathways for prenatal diagnosis of fetal anomalies using prenatal ES, from referral for testing until birth outcome. This will be done for each of the seven GLHs and the optimal pathway, and will be based on data collected during Workstream-1.
2) Using the linked FMU outcomes/National Congenital Anomaly and Rare Disease Registration Service data collected during Workstream-3 we will plot the movement of pregnant women through each of the pathways. We will extract information on the numbers of women undergoing different tests, the numbers and type of fetal anomalies identified, the number of follow-up contacts related to testing, and pregnancy outcomes.
3) We will identify the unit costs associated with the main cost components of the identified pathways. These will be obtained from the micro-costing, supplemented with other unit costs from the GLHs, and published and other routinely available sources.
4) We will calculate the NHS costs associated with each pathway, by applying the unit costs associated with each item in the pathway from stage 3 with the numbers of women incurring that cost based on the data at stage 2.
5) We will calculate the financial costs to parents and families from the different pathways using parent questionnaires developed following parent interviews in Workstream-2.
6) We will undertake a cost consequences analysis comparing the NHS and family costs of each pathway against the consequences, as delineated in Workstream-3 (e.g., diagnostic yield, birth outcome).
7) We will use our analysis to assess the expected budget impact to the NHS of introducing prenatal ES, based on the mean costs per woman tested and projections of the expected numbers of women tested by prenatal ES nationally.
8) We will identify the main sources of uncertainty in our analyses and undertake sensitivity to explore the impacts of this uncertainty.

### Integration of findings

Using an approach of simultaneous triangulation ^39^, we will draw together data collected in the qualitative analyses of the service (Workstream-1), stakeholder perspectives (Workstream-1, Workstream-2 and Workstream-4), quantitative analyses of clinical outcomes (Workstream-3), ethical analysis (Workstream-4), and the economics analysis (Workstream-5) to identify the main features of the prenatal ES service nationally and points of local variation. We will also conduct workshops to determine the key features of an optimal care pathway from a service delivery, patient and professional perspective. Through this process we will define current service provision, identify the facilitators and barriers to optimal service delivery and highlight key lessons to inform future models of service provision and will produce recommendations for best practice.

### Ethical approval and consent to participate

Our research will be conducted in accordance with the UK Policy Framework For Health and Social Care Research which sets out the principles of good practice in the management of research. Qualitative and quantitative data for this research will be collected in a range of settings, and participants will include parents, health professionals and policy makers. Research involving parents has been reviewed by the Health Research Authority (HRA) and an NHS Research Ethics Committee (East of Scotland Research Ethics Service REC 1): “Parental views and experiences of prenatal exome sequencing” 21/ES/0073. Research involving professionals has been classified as a Service Evaluation, not requiring research ethics committee approval, by the HRA. The service evaluation has been registered with the R&D office at Great Ormond Street Hospital for Children NHS Foundation Trust.

Invitations to parents to take part in an interview or workshop will be sent after the parents have been offered ES and have made their decision to accept or decline testing so that the research does not impact on parents’ decision-making about prenatal testing. For interviews with parents and professionals, the potential participants will be given a participant information sheet describing the study, what participation involves, confidentiality and plans for data protection and data storage and written or verbal (recorded) consent will be obtained. For the surveys, returning a completed survey will be taken as implied consent to participate.

### Study status

The study commenced on October 1 ^st^ 2020. The study is currently open for recruitment.

### Study registration

The EXPRESS study was prospectively registered with the Research Registry (researchregistry6138).

### Dissemination plan

Dissemination will be both formative, as we will feed back findings as the study proceeds, and summative. Our strategy for engagement, formative feedback and dissemination includes:

•   Workshops with professionals from a range of backgrounds.

•   Progress reports shared at a national level with the NHS Genomics Laboratory Hub Partnership Board and professional bodies, such as the Joint Committee on Genomics in Medicine and the British Maternal and Fetal Medicine Society.

•   Peer reviewed publications.

•   Presentations at national and international conferences.

•   Plain language summaries of findings, written with the help of the PPIEP Advisory Group, will be disseminated to parent and patient networks via meetings, newsletters, social media and the EXPRESS study website.

•   A policy report that will describe the facilitators and barriers to optimal service delivery and deliver recommendations for best practice.

## Discussion

The EXPRESS study will inform the evolution of a prenatal ES service that delivers equity of access and high standards of care across England with an associated improvement in prenatal diagnostic services and benefits for parents. Our findings will be shared with key stakeholders on a regular basis throughout the course of the study to facilitate improvements in service delivery, and identify future evaluation and research needs. This work will also be an exemplar for evaluating other aspects of the NHS GMS; for example, recommendations about how best to optimise communication between clinical genetics, laboratories and non-genetic specialists will be transferable, as well as recommendations around supporting equity of access and inclusion of diverse population groups. As the NHS is an early adopter of prenatal ES, findings may be useful to others internationally as they implement similar services. As our research will commence within the first year of the prenatal ES service, we anticipate generating lessons for the GLHs within the timeframe of the study.

A key strength of the research is our mixed-methods approach and engagement with stakeholders from a range of backgrounds. The duration of the study means that we will be responding to themes arising in the case studies and will allow us to study developments within the service and strategic responses to issues in the service. As previously noted, PPIEP will be embedded throughout the study. There are, however, some potential limitations. The multi-site nature of the study and having several different workstreams will require GLHs to be highly engaged with the research. In addition, as the study is focused on the implementation of prenatal ES within the NHS, a national healthcare service that is unique in many ways, some findings may not be directly generalisable to healthcare systems in other countries. However, we do anticipate that many challenges will be common across countries and lessons from the study will be transferable to other settings. Adapting to challenges created by the Covid-19 pandemic will impact our evaluation. In particular, approaches to data collection may be amended. Working remotely and offering interviews by phone or video call will be used if needed. This approach reflects how health services are adapting to Covid-19 with the use of virtual appointments, but we do recognise that virtual appointments can be a barrier for some people. Comparison of telephone and face-to-face interviews indicates data quality and richness is similar ^40^ and participants reportedly value the practical ease of being interviewed by telephone and some can feel more comfortable when discussing sensitive topics ^41, 42^. However, privacy needs and access to technology need consideration and may necessitate in person interviews in some cases.

## Data availability

### Underlying data

No data are associated with this article. Anonymised data underlying the results will be made accessible through the UCL Data Repository and a DOI will be referenced in research publications. Data will be made available under the terms of the Creative Commons Attribution 4.0 (CC BY 4.0).

### Reporting guidelines

University College London: SRQR Checklist for the Optimising EXome PREnatal Sequencing Services (EXPRESS) study, https://doi.org/10.5522/04/17277386 ^43^.

Data are available under the terms of the Creative Commons Attribution 4.0 International license (CC-BY 4.0).
