# Supplementary material for: Optimising Exome Prenatal Sequencing Services (EXPRESS): a study protocol to evaluate rapid prenatal exome sequencing in the NHS Genomic Medicine Service
Source: NIHR Open Res. 2022 Jul 18;2:10. Originally published 2022 Feb 3. [Version 2] doi: 10.3310/nihropenres.13247.2 (PMC7613246; doi:10.3310/nihropenres.13247.2)
Supplement: Peer review history

# Reviewer report: Reviewer response for version 2

**DOI:** 10.3310/nihropenres.14410.r28710

**Published:** 2022-07-25

**Authors:** Jane Halliday

**Affiliations:** 1 Reproductive Epidemiology group, Murdoch Children's Research Institute, Parkville, Vic, Australia

I have reviewed this new version and the response to my comments, and I am satisfied that they have attended to them very carefully. I only have one very minor comment:

 When defining the Objectives, it is not necessary to have Workstream number listed within each objective as, at this stage, what this means is unknown. When describing each Workstream, perhaps note which objective (A-G) is relevant. However, maybe this is not necessary as the section on Integration of Findings is now so clear. Just a thought

Is the study design appropriate for the research question?

Yes

Is the rationale for, and objectives of, the study clearly described?

Yes

Are sufficient details of the methods provided to allow replication by others?

Partly

Are the datasets clearly presented in a useable and accessible format?

Not applicable

Reviewer Expertise:

Epidemiology, prenatal genetics

I confirm that I have read this submission and believe that I have an appropriate level of expertise to confirm that it is of an acceptable scientific standard.

# Reviewer report: Reviewer response for version 1

**DOI:** 10.3310/nihropenres.14363.r28595

**Published:** 2022-05-17

**Authors:** Vaidehi Jobanputra

**Affiliations:** 1 New York Genome Center, New York, NY, USA

This is an important study the NHS has embarked to evaluate the prenatal exome sequencing service. The NHS in England is the first large national healthcare system to systematically embed genome and exome sequencing in routine clinical care. The overall study design will deliver high throughput and high-quality genomic testing with equity of access for patients across the NHS and collect potential data for variation in referrals, uptake, diagnostic rates, parent and provider expectations, costs, and technical considerations of genomic reporting in a prenatal setting.

 The 3-year prospective evaluation of prenatal ES in the NHS GMS will analyze the national implementation of prenatal and determine an optimal pathway that maximizes benefits for parents while optimizing use of NHS resources. This research will identify ethical and practical issues and highlight any unintended consequences of the new testing modality.

 The research study design is based on a framework developed in previous studies of major system innovation, which is well thought through. A Steering Committee with academic, professional, and patient and public involvement, will oversee the evaluation and provide guidance through regular interactions with the research team throughout the study. The study has 7 objectives clearly defined with the nationwide setting and will look at provisions of prenatal ES across England. The study team has identified Phases of the study, data collection and analysis for each phase is also described. The strengths and limitations are recognized by the study team. I am looking forward to seeing the results of this innovative study which will inform the implementation of prenatal ES nationally and some aspects may be informative internationally.

Is the study design appropriate for the research question?

Yes

Is the rationale for, and objectives of, the study clearly described?

Yes

Are sufficient details of the methods provided to allow replication by others?

Yes

Are the datasets clearly presented in a useable and accessible format?

Yes

Reviewer Expertise:

Genomics, Reproductive science, Molecular Diagnostics, Prenatal Diagnosis

I confirm that I have read this submission and believe that I have an appropriate level of expertise to confirm that it is of an acceptable scientific standard.

# Response

**Published:** 2022-06-25

**Authors:** Melissa Hill

**Affiliations:** Great Ormond Street Hospital for Children, UK

Thank you for your positive feedback on the manuscript.

# Reviewer report: Reviewer response for version 1

**DOI:** 10.3310/nihropenres.14363.r28474

**Published:** 2022-03-01

**Authors:** Jane Halliday

**Affiliations:** 1 Reproductive Epidemiology group, Murdoch Children's Research Institute, Parkville, Vic, Australia

This important research is obviously well underway, but this was not clear to me until I got to page 10. I wrote the following before realising this:

 EXPRESS is described as a 3-year prospective evaluation of prenatal ES in the NHS GMS. This is confusing as on page 4 it says that prenatal ES was implemented nationally in the NHS GMS in Oct 2020, yet in other places the implication is that this has not begun, and the evaluation will be on the 1 ^st^ 6 months, 18 months etc.

- See 1 ^st^ para on page 5 – ‘research will commence in the first year of the prenatal ES service’.
- Also, on page 6, phase 1 of Workstream 1 covers the 1 ^st^ 6 months, while phase 2 covers 6-18 months.
- Also, on page 7 under Phase 3, ‘As the prenatal ES service is entirely new to the NHS, there is no baseline,..”

This needs clarification – how does EXPRESS fit into the existing service? Has it already begun?

**When I reached page 10, I saw that the study began in Oct 2020, so this protocol is retrospective. **Does the protocol need to reflect this somehow with use of past tense when appropriate? It refers to COVID-19 in the Discussion, so this is a contemporary issue.

**Use of Figure 1:** The way this was referred to in the text could have been clearer; why were details relating to the 4 boxes on the LHS provided, but no other sections? Does the phrase ‘developing and agreeing new service models’ = ‘decisions on care pathways’? This Figure is not referred to elsewhere.

**Aligning of Objectives and Workstreams** was not always done. For instance, where are the health professional education and information needs (part of Objective C) studied? 

**WS1:** Phase 1, section 2): ‘key staff’ = ‘professionals’ ? What is the difference between 2) and 3) interviewees – those establishing the service versus those delivering the service? This should be made clearer and not left until a few paragraphs later where there is a rather vague description of recruitment of ‘professionals’, somewhat a repeat of phase 1, section 3).

 How will the quantitative surveys mentioned in WS 1, phase 2, examine ‘referral pathways and patient flow..’ and then be summarised as frequencies (see last sentence of data analysis)?

**WS2: **Selection/recruitment bias? 1 ^st^ paragraph says 'participants will be purposefully sampled to ensure…'. The next paragraph says parents who have accepted or declined ES will be identified and a letter sent to potential participants (I presume this includes those who declined) who will then have to contact the research team themselves if they decide to participate. The same process is used for those offered ES and those with previous fetal anomaly. How will bias be recognised and dealt with? 

**WS3:** Data quality. It seems imperative that outcome data quality is high, so a reference to the quality is needed. Is there complete ascertainment of outcomes of all types?

 The dot points relating to the descriptive analysis are very simplistic, especially the 4 ^th^ where covariates and outcomes are mixed up. What is the ‘source of referral’ – is this the GLH? If not, is that to be something also collected under dot point 2?

**WS4**: Information will be used from WS1 interviews with 2-3 professionals per GLH. Then there will be 3-4 Workshops, also with people from the GLHs and clinical services. This all sounds rather vague, but maybe that’s OK – could they be the same people? Will professionals and parents be in the same workshops?

**WS5**: This was clear and plenty of detail supplied.

 The **Integration of findings** section (relating to Objective G, I presume) also has workshops, but who with etc? I’m not sure if such lack of detail is acceptable for a protocol paper like this, but I am left feeling that the evaluation is very open-ended. Maybe refer back to Figure 1 to help bring it all together.

Is the study design appropriate for the research question?

Yes

Is the rationale for, and objectives of, the study clearly described?

Yes

Are sufficient details of the methods provided to allow replication by others?

Partly

Are the datasets clearly presented in a useable and accessible format?

Not applicable

Reviewer Expertise:

Epidemiology, prenatal genetics

I confirm that I have read this submission and believe that I have an appropriate level of expertise to confirm that it is of an acceptable scientific standard, however I have significant reservations, as outlined above.

# Response

**Published:** 2022-06-25

**Authors:** Melissa Hill

**Affiliations:** Great Ormond Street Hospital for Children, UK

Thank you for your thoughtful and constructive comments. We have revised the manuscript and hope that your comments have been addressed to your satisfaction.

**Reviewer 1**

**This important research is obviously well underway, but this was not clear to me until I got to page 10. I wrote the following before realising this: EXPRESS is described as a 3-year prospective evaluation of prenatal ES in the NHS GMS. This is confusing as on page 4 it says that prenatal ES was implemented nationally in the NHS GMS in Oct 2020, yet in other places the implication is that this has not begun, and the evaluation will be on the 1 ^st^ 6 months, 18 months etc. See 1 ^st^ para on page 5 – ‘research will commence in the first year of the prenatal ES service’. Also, on page 6, phase 1 of Workstream 1 covers the 1 ^st^ 6 months, while phase 2 covers 6-18 months. Also, on page 7 under Phase 3, ‘As the prenatal ES service is entirely new to the NHS, there is no baseline,..” This needs clarification – how does EXPRESS fit into the existing service? Has it already begun?**

**When I reached page 10, I saw that the study began in Oct 2020, so this protocol is retrospective.** **Does the protocol need to reflect this somehow with use of past tense when appropriate? It refers to COVID-19 in the Discussion, so this is a contemporary issue.**

 We agree that the start date of EXPRESS matching the start date of the prenatal ES service was not made clear until part way through the manuscript. We have added a sentence to the abstract and to the last paragraph of the introduction to make it clear upfront that EXPRESS started at the same time as the prenatal ES service. We have also changed the tense throughout when referring to the start of the service or the start of EXPRESS.

**Use of Figure 1:** **The way this was referred to in the text could have been clearer; why were details relating to the 4 boxes on the LHS provided, but no other sections? Does the phrase ‘developing and agreeing new service models’ = ‘decisions on care pathways’? This Figure is not referred to elsewhere.**

 Thank you for picking up the discrepancy in the wording between the figure and the text. We have changed the text describing the key elements of the framework for major systems innovation to match the wording in the figure. We have also added further information in the text about the framework (including additional references to the figure) and how it will be applied in our analysis to the sections on Study Design, Workstream-1 - Data Collection and Analysis and Integration of Findings.

 This feedback prompted us to add more detail about how the analysis of the three separate phases in worksteam-1 will be conducted. As we worked through this revision, we made the decision to remove Normalisation Process Theory from our planned analysis framework to focus on the framework for major systems innovation and the MRC guidance for process evaluations. As such we have removed the reference to Normalisation Process Theory throughout the manuscript.

**Aligning of Objectives and Workstreams** **was not always done. For instance, where are the health professional education and information needs (part of Objective C) studied?**

 To address this point we have added the relevant workstream(s) to each of objectives listed in the Specific Objectives. Training and education needs of professionals will be addressed in workstream-1 through the interviews and surveys with professionals – we have added a reference to this in the description of workstream-1.

**WS1:** **Phase 1, section 2): ‘key staff’ = ‘professionals’ ? What is the difference between 2) and 3) interviewees – those establishing the service versus those delivering the service? This should be made clearer and not left until a few paragraphs later where there is a rather vague description of recruitment of ‘professionals’, somewhat a repeat of phase 1, section 3).**

 We have added additional detail to distinguish between the types of professionals we will interview in our national level interviews – staff involved at a national level in deciding to implement the service and who developed the national guidance on service delivery (point 2) and in our local level interviews - the staff on the ground at each GLH who are delivering the service in their daily clinical practice (point 3).

**How will the quantitative surveys mentioned in WS 1, phase 2, examine ‘referral pathways and patient flow..’ and then be summarised as frequencies (see last sentence of data analysis)?**

 We have changed the wording of the sentence referring to frequencies to be clear that descriptive statistics will be used to analyse the survey data. We have also included additional detail about the analysis plans for the three phases of data collection described in workstream-1. This includes a description of how the summarising of the qualitative and quantitative data into case study templates will allow service components and care pathways to be categorised into typologies for comparison within and between GLHs.

**WS2:** **Selection/recruitment bias? 1 ^st^ paragraph says 'participants will be purposefully sampled to ensure…'. The next paragraph says parents who have accepted or declined ES will be identified and a letter sent to potential participants (I presume this includes those who declined) who will then have to contact the research team themselves if they decide to participate. The same process is used for those offered ES and those with previous fetal anomaly. How will bias be recognised and dealt with?** 

 We agree that there is the potential for selection bias in the parent interviews and also in the professional interviews/survey where purposeful sampling will also be used. Letters of invitation to take part in an interview will be sent to parents who accepted prenatal ES testing and also to those who declined. We have added an additional step in our recruitment procedure to engage parents in the study, whereby the local clinical team will call the potential participants to discuss the study if they have not responded to the initial letter of invitation. To reduce the risks of selection bias for the professional survey and interviews we are approaching key contacts, such as the clinical geneticists who act as service leads for their region, so that we target the most appropriate people in each GLH with our study invitations. We have added a sentence to the discussion to acknowledge the risk of selection bias. The risk of selection bias will be acknowledged and discussed in the limitations section of any resulting publications.

**WS3:** **Data quality. It seems imperative that outcome data quality is high, so a reference to the quality is needed. Is there complete ascertainment of outcomes of all types?**

**The dot points relating to the descriptive analysis are very simplistic, especially the 4 ^th^ where covariates and outcomes are mixed up. What is the ‘source of referral’ – is this the GLH? If not, is that to be something also collected under dot point 2?**

 We have revised the section describing workstream-3, adding further information about data quality, clarified the ‘source of referral’, and moved information related to data collection to a sub-section titled “Data collection and analysis”. We have also delineated the covariates and outcomes by adding a separate bullet point for each one.

**WS4** **: Information will be used from WS1 interviews with 2-3 professionals per GLH. Then there will be 3-4 Workshops, also with people from the GLHs and clinical services. This all sounds rather vague, but maybe that’s OK – could they be the same people? Will professionals and parents be in the same workshops?**

 We have added additional detail about the planned workshops. Separate workshops for professionals and parents will be held. We hope that holding workshops specifically for parents will help to facilitate an open discussion that is focussed on the issues that are most important to parents. Parents will be invited through patient groups (e.g. from ARC, UNIQUE and Genetic Alliance UK) and through NHS maternity services. For the workshops with professionals it is possible that some professionals will attend more than one workshop as invitations will be advertised broadly and no restrictions on attendance will be applied. This potential for bias will be discussed in any publications that arise.

**WS5** **: This was clear and plenty of detail supplied.**

 Thank you.

**The Integration of findings section (relating to Objective G, I presume) also has workshops, but who with etc? I’m not sure if such lack of detail is acceptable for a protocol paper like this, but I am left feeling that the evaluation is very open-ended. Maybe refer back to Figure 1 to help bring it all together.**

****

 As suggested, we have added a paragraph to the section on Integration of findings to highlight that integration of the data from all workstreams will focus on addressing our study objectives and be underpinned by the conceptual framework of major system innovation which is described in Figure 1.

 We have added more detail about who will be invited and the purpose and content of the proposed workshops.
